# The selective alpha7 nicotinic acetylcholine receptor agonist AR-R17779 does not affect ischemia–reperfusion brain injury in mice

**DOI:** 10.1042/BSR20210736

**Published:** 2021-06-11

**Authors:** Maria E. Hammarlund, Vladimer Darsalia, Filip Mjörnstedt, Bagmi Pattanaik, Carina Mallard, Eridan Rocha-Ferreira, Cesare Patrone, Maria E. Johansson

**Affiliations:** 1 Department of Physiology, Institute of Neuroscience and Physiology, Sahlgrenska Academy, University of Gothenburg, Gothenburg, Sweden; 2 Department of Clinical Science and Education, Södersjukhuset, Internal Medicine, Karolinska Institutet, Stockholm, Sweden; 3 Department of Obstetrics and Gynecology, Institute of Clinical Sciences, Sahlgrenska Academy, University of Gothenburg, Gothenburg, Sweden

**Keywords:** α7nAChR, agonists, alpha7 nicotinic acetylcholine receptor, Chrna7, inflammation, ischemia reperfusion injury

## Abstract

Inflammation plays a central role in stroke-induced brain injury. The alpha7 nicotinic acetylcholine receptor (α7nAChR) can modulate immune responses in both the periphery and the brain. The aims of the present study were to investigate α7nAChR expression in different brain regions and evaluate the potential effect of the selective α7nAChR agonist AR-R17779 on ischemia–reperfusion brain injury in mice. Droplet digital PCR (ddPCR) was used to evaluate the absolute expression of the gene encoding α7nAChR (*Chrna7)* in hippocampus, striatum, thalamus and cortex in adult, naïve mice. Mice subjected to transient middle cerebral artery occlusion (tMCAO) or sham surgery were treated with α7nAChR agonist AR-R17779 (12 mg/kg) or saline once daily for 5 days. Infarct size and microglial activation 7 days after tMCAO were analyzed using immunohistochemistry. *Chrna7* expression was found in all analyzed brain regions in naïve mice with the highest expression in cortex and hippocampus. At sacrifice, white blood cell count was significantly decreased in AR-R17779 treated mice compared with saline controls in the sham groups, although, no effect was seen in the tMCAO groups. Brain injury and microglial activation were evident 7 days after tMCAO. However, no difference was found between mice treated with saline or AR-R17779. In conclusion, α7nAChR expression varies in different brain regions and, despite a decrease in white blood cells in sham mice receiving AR-R17779, this compound does not affect stroke-induced brain injury.

## Introduction

Stroke is the third leading cause of death [1] and the leading cause of disabilities [2] in the western world, with ischemic stroke accounting for 80% of all stroke incidents. Despite increasing knowledge about the molecular mechanisms involved in stroke pathology, pharmacological treatment is still limited to tissue plasminogen activators (tPA) that act by breaking down clot formation [3]. Treatment with tPA can indeed be effective, but early administration is crucial and the treatment window is set to 4.5 h after onset of stroke. Still, only one in seven patients show reduced disability even when tPA is administered 3.0–4.5 h after stroke onset [3]. Therefore, there is a great need for alternative treatment strategies.

During an ischemic event, blood flow is disrupted and the tissue suffers from oxygen and nutrient deficit, resulting in several biochemical events eventually leading to neuronal cell death at the center of the infarct [4]. The degree of brain injury depends on several factors including time of ischemia and the ability of the brain to recover. Neuronal cell death causes danger-associated molecular patterns (DAMPs) to be released, initiating an inflammatory response where both the innate and adaptive immune system are activated [5]. Microglia, the main immune cell of the central nervous system (CNS), is a key mediator in regulating homeostasis and immunosurveillance in the CNS. In pathological conditions, e.g., stroke, microglia rapidly become activated to present antigens and release cytokines and other inflammatory mediators, which may contribute to the brain injury [6]. Approaches targeting inflammation have been tested to treat stroke-induced injuries, yet translation to the clinic has been unsuccessful [5].

In the central nervous system, the alpha 7 nicotinic acetylcholine receptor (α7nAChR) is involved in the regulation of neuronal plasticity [7] and neuroprotection by improving cell survival [8]. Moreover, the α7nAChR has been studied as a potential pharmacological target for neurodegenerative diseases such as Alzheimer’s disease [9], Parkinson’s disease [10] and Schizophrenia [11]. Although the α7nAChR is known to be expressed in neurons [7], astrocytes [12,13] and microglial cells [14], α7nAChR expression patterns are not well established in different brain regions. Interestingly, we and others have demonstrated that α7nAChRs can modulate inflammatory responses in the periphery in several different experimental models [15–17]. Furthermore, α7nAChR stimulation can modulate microglia responses by reducing the production of proinflammatory cytokines *in vitro* [14,18–20] and can reduce brain injury in hemorrhagic stroke [21]. However, data on the effect of α7nAChR stimulation in ischemia–-reperfusion injury is sparse. Over the years, several different agonists for the α7nAChR have been developed, most of them being partial agonists [22], but also full agonists such as AR-R17779. The AR-R17779 compound is blood–brain penetrable [23], with a high efficacy of 96% [24] and compared with other α7nAChR agonists, highly specific to the α7nAChR [24–26]. Thus, the present study aimed to investigate α7nAChR expression patterns in different brain regions affected by ischemia–reperfusion injury in mice and evaluate the role of the selective α7nAChR agonist AR-R17779 on ischemia–reperfusion brain injury.

## Methods

### Animals

Adult male C57BL/6Jrj mice were purchased from Janvier Labs (Janvier Labs, Le Genest-Saint-Isle, France). The animals were housed at the Experimental Biomedicine, University of Gothenburg, with free access to food and water and kept at a 12/12 h light/dark schedule. Animals were acclimated for a minimum of 5 days before the experiment. All experiments in the present study were approved by the Regional Animal Ethics Committee at the University of Gothenburg, in accordance with the European Communities Council Directives of 22 September 2010 (2010/63/EU).

### Absolute quantification of *Chrna7* expression

Naïve, male C57BL/6Jrj mice (27 weeks old, *n*=6) were killed by an overdose of pentobarbital (i.p., Apoteket AB, Stockholm, Sweden) and perfused intracardially via apex with physiological saline. Brains were dissected and cortex, striatum, hippocampus and thalamus were isolated, snap frozen and kept at −80°C until further analysis.

mRNA from hippocampus, striatum, thalamus and cortex was extracted using the RNAeasy® Lipid Tissue Mini kit (Qiagen GmbH, Hilden, Germany) as previously described [16], followed by assessment of RNA concentration using NanoDrop (NanoDrop Products, DE, US). Reverse transcription was performed using the QuantiTect Reverse Transcription Kit (Qiagen GmbH, Hilden, Germany) according to the manufacturer’s protocol. The expression of *Chrna7* in different brain regions was determined with droplet digital PCR (ddPCR) utilizing EvaGreen (Bio-Rad Laboratories, Hercules, CA, U.S.A.). In brief, a reaction mix was prepared by mixing 2X QX200™ ddPCR™ EvaGreen Supermix (Bio-Rad Laboratories, Hercules, CA, U.S.A.), forward and reverse primers, RNase free-water and cDNA. The reaction mix was used to generate droplets with the QX200 droplet generator (Bio-Rad Laboratories, Hercules, CA, U.S.A.). After droplet generation, samples were transferred to a 96-well plate, sealed and amplified in a T100 Thermal Cycler (Bio-Rad Laboratories, Hercules, CA, U.S.A.) with following settings: 95°C for 5 min, 40 cycles of 96°C for 30 s and 58.8°C for 1 min, 4°C for 5 min followed by 90°C for 5 min. A ramp rate of 2°C/s was used for all steps. Finally, the samples were analyzed in the QX200 Droplet Reader (Bio-Rad Laboratories, Hercules, CA, U.S.A.) and the results, including positive and negative droplets, were analyzed with QuantaSoft Analysis Pro™ Bio-Rad Laboratories, Hercules, CA, U.S.A.). Primers for *Chrna7* (Forward: 5′ to 3′ GCATGAAGAGGCCGGGAGAGGACAAG, Reverse: 5′ to 3′ GTGTGTGGTCGTTTGGCCTGCTCCC) were designed in-house and purchased from Invitrogen (Carlsbad, CA, U.S.A.).

### Cerebral ischemia and agonist treatment

Ischemia reperfusion injury was carried out using the intraluminal transient middle cerebral artery occlusion (tMCAO) model first developed by Koizumi [27], and modified by Longa [28]. In brief, mice (12 weeks old, 22–30 g) were anesthetized with isoflurane (initially 5% and then kept at 2.5%) (Attane vet, VM Pharma AB, Stockholm, Sweden). An incision was made in the neck and the external carotid artery (ECA) was isolated and permanently ligated using a 7/0 silk suture. The common carotid artery (CCA) was temporarily ligated and clamped at the bifurcation of the CCA. A small incision was made in the ECA and a silicone-rubber coated nylon monofilament (diameter 0.18 mm, coating length 5–6 mm; Doccol Incorporation, Sharon, Massachusetts) was inserted through the ECA into the internal carotid artery (ICA) until it reached the middle cerebral artery (MCA), and secured with a temporary ligation. The ICA was occluded for 30 min during which the mouse was allowed to wake up. After 30 min occlusion, the filament was removed during anesthesia, the ECA permanently ligated, and the blood flow restored in the CCA. The incision was closed with 5/0 silk suture and lidocaine ointment applied topically onto the closed incision. Temgesic (0.1 mg/kg s.c., Indivior Europe Limited, Dublin, Ireland) was given subcutaneously and mice were allowed to wake up in a heated cage. Control mice underwent sham surgery where the filament was inserted in the ECA, but instantly removed. Sham mice were also anesthetized a second time.

One hour after surgery, mice were randomized into receiving α7nAChR agonist AR-R17779 (55 µmol/kg, 12 mg/kg i.p., Tocris, Abingdon, UK; MCAO *n*=17, Sham *n*=7) or saline (0.9%, i.p., Apoteket AB, Sweden; MCAO *n*=15, Sham *n*=6) in volumes according to body weight. The AR-R17779 dose was chosen based on previous studies [29,30]. First treatment was given one hour post-surgery and thereafter once daily for the following four days. Health status and weight were checked daily from surgery until euthanasia. The surgeries were carried out at two different time points with two different operators VD (*n*=18) and MEH (*n*=27). One mouse from the MCAO saline group was excluded due to lack of brain injury. One mouse in the MCAO saline and three in the MCAO agonist group were excluded due to severe complications after surgeries and/or sudden death.

### Tissue collection and preparation

Seven days following surgery, mice were killed by an overdose of pentobarbital (i.p., Apoteket AB, Sweden). Blood was collected from the right ventricle into EDTA tubes for hematology analysis and serum saved for subsequent analysis. Mice were perfused intracardially with saline followed by Histofix (Histolab, Askim, Sweden). Spleens were ligated before perfusion, isolated and weighed. Brains were isolated, weighed and stored in Histofix for a minimum of 48 h. Fixed brains were kept in 70% ethanol for 1.5 h prior to dehydration. Following dehydration, brains were embedded in paraffin. Brains were sectioned in a microtome in 8 µm thick, coronal sections, in caudal-rostral direction starting at the interaural line. Serial sections were collected with 400 µm between each section and mounted onto Superfrost plus slides (VWR, Radnor, PA, U.S.A.).

### Immunohistochemistry

Immunohistochemistry was performed as previously described [31]. In brief, sections were de-paraffinized with a graded series of xylene and ethanol. Antigen retrieval was done by boiling the sections in citrate buffer (0.01 M, pH 6) for 10 min. Sections were thereafter incubated with H_2_O_2_ (3%) for 10 min for blocking of peroxidases and blocked with normal horse serum (Vector Laboratories, Burlingame, CA, U.S.A.) for 30 min. Sections were incubated with primary antibodies against microtubule-associated protein 2 (MAP2; clone HM-2, 1:1000; Sigma-Aldrich, St. Louis, MO, U.S.A.) or ionized calcium-binding adapter molecule 1 (Iba-1; Cat no 019-19741, Fujifilm Wako Chemicals U.S.A. Corporation, Richmond, VA, U.S.A.) overnight at 4°C. The sections were then incubated with the corresponding biotinylated secondary antibody (Vector Laboratories, Burlingame, CA, U.S.A.) for 60 min at room temperature. Peroxidase staining was detected using Vectastain ABC Elite (Vector Laboratories, Burlingame, CA, U.S.A.) with 3,3-diaminobenzidine (0.5 mg/ml) enhanced with ammonium nickel sulfate (15 mg/ml), β-D-glucose (2 mg/ml), ammonium chloride (0.4 mg/ml), and β -glucose oxidase (0.01 mg/ml; all chemicals purchased from Sigma-Aldrich, St. Louis, MO, U.S.A.). Sections were dehydrated with graded series of ethanol and xylene and mounted using Pertex Histofix (Histolab, Askim, Sweden).

### Blood analysis

Whole blood samples collected in EDTA tubes at the time of euthanasia were analyzed using Vetscan HM5 (Abaxis, Union City, CA, U.S.A.) to analyze blood hematology parameters. Serum samples were collected by centrifugation of whole blood (10000 ***g*** for 5 min) and the supernatant was collected and stored at −80°C until analysis. Serum samples were analyzed for cytokine levels by Luminex (Bio-Plex Pro™ Mouse chemokine Panel 33-plex; Bio-Rad Laboratories) according to the manufacturer’s protocol as previously described [16].

### Brain injury evaluation

MAP2-stained sections were analyzed to quantify grey matter injury [32–34]. For quantification of infarct size, every 50th section throughout the brain was stained for MAP2. Images were captured using an Olympus light microscope with 1.25× magnification and analyzed using ImageJ (version 1.51j8, http:/imageJ.nih.gov/ij, National Institutes of Health, U.S.A.). Infarct size was calculated by subtracting the ipsilateral MAP2-stained area from the contralateral and expressing the difference as percentage of the stained contralateral area [32]. Quantifications were made for the whole hemisphere and for cortex specifically. All quantifications were performed in a blinded manner.

Iba-1 stained sections were analyzed by phenotype scoring, using a modified version of a previously described scoring method [35,36]. Briefly, affected brain regions (striatum, upper and lower cortex, cerebral peduncle, globus pallidus and substantia nigra) were given a score 0-4, based on microglial phenotype and accumulation (Supplementary Figure S1). Each region was scored using a light microscope (Olympus) with three 20× magnified pictures at each level. A mean score for each region was calculated at each level, and then a mean for the region throughout the different levels in which the region was present.

### Statistical analysis

Data were tested for normality with Shapiro–Wilk test and statistical methods were chosen accordingly. ddPCR data were analyzed with Kruskal–Wallis test followed by Dunn’s multiple comparisons test for post hoc analysis. Hematology parameters were analyzed with unpaired *t*-test. Serum protein concentrations were analyzed with Kruskal–Wallis followed by Bonferroni correction for multiple comparisons. MCAO surgeries were performed by two different operators at two different time points; therefore, infarct size were analyzed with two-way ANOVA with operators as co-variant. Body weight change, brain weight and normalized spleen weight were analyzed with one-way ANOVA, followed by Sidak’s multiple comparisons test for post hoc analysis. The statistical analyses were carried out with the GraphPad Prism software (Version 8.1.2, La Jolla, CA, U.S.A.) or IBM SPSS (IBM SPSS Statistics for Windows, Version 25.0. Armonk, NY, U.S.A.). Data are expressed as mean ± SEM. *P*<0.05 was considered to be statistically significant. The number of mice per group and statistical details are stated in the figure legends and tables.

## Results

### Distinct *Chrna7* expression patterns in mouse brain regions

**Figure 1:**
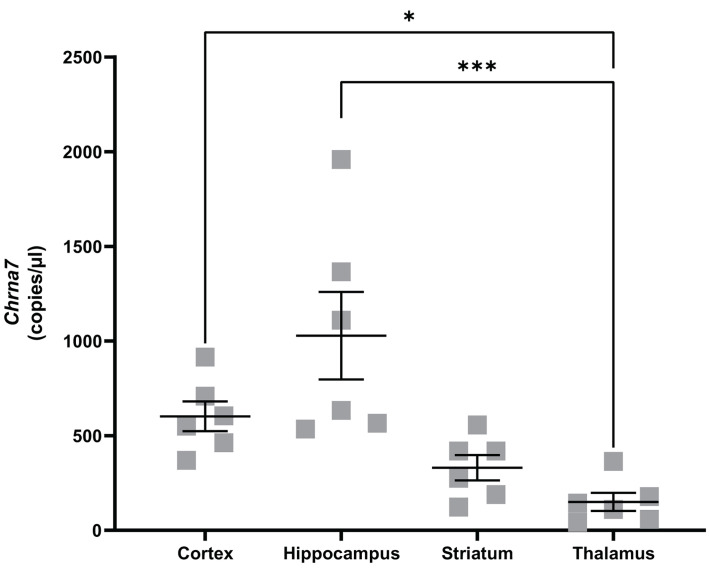
*Chrna7* mRNA expression levels are significantly higher in hippocampus and cortex compared to thalamus of naïve mice Absolute quantification of *Chrna7* mRNA expression using droplet digital PCR (ddPCR) in mouse brain regions (cortex, hippocampus, striatum and thalamus) of naïve, adult mice (*n*= 6). Data were analyzed with Kruskal–Wallis test followed by Dunn’s multiple comparisons test. Data are expressed as mean ± SEM. *P<0.05; **P<0.01, ***P<0.001

Using naïve mice, *Chrna7* expression patterns were investigated in different brain regions (cortex, striatum, hippocampus, and thalamus) known to be affected by the transient middle cerebral artery occlusion (tMCAO) model. Absolute quantification of *Chrna7* expression was carried out using ddPCR. *Chrna7* expression was detected in all the analyzed brain regions, with significantly higher expression in cortex (Kruskal–Wallis, *P*=0.0007, Dunn’s multiple comparisons test, *P*=0.022) and hippocampus (*P*=0.00088) compared with thalamus (Figure 1).

### α7nAChR agonist AR-R17779 does not affect stroke-induced brain damage in the tMCAO model

**Figure 2:**
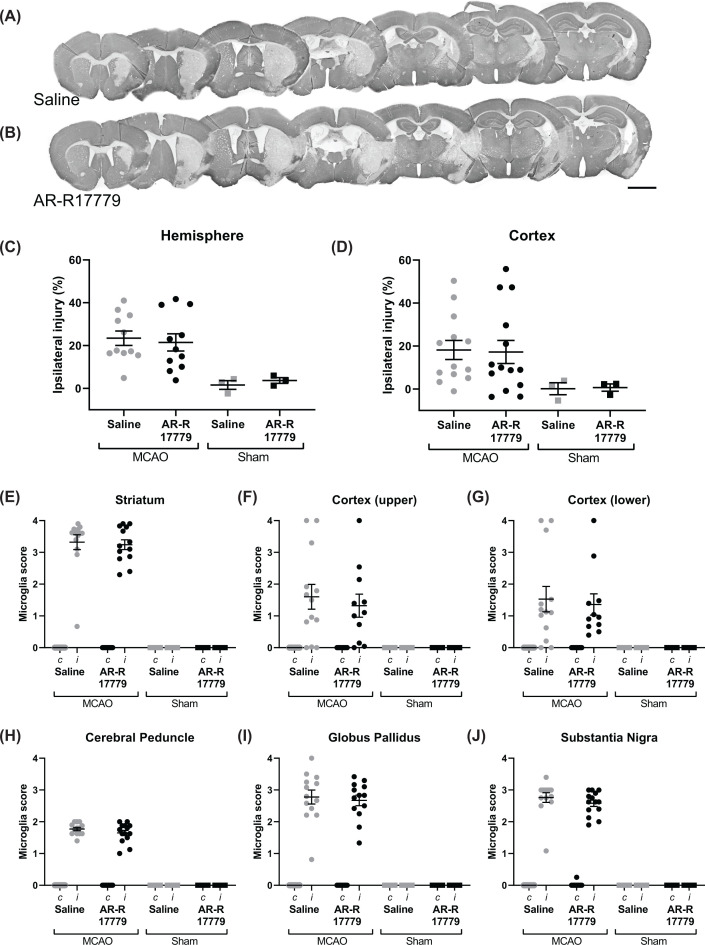
α7nAChR agonist AR-R17779 does not affect infarct size or microglial activation in stroke-induced brain injury Mice undergoing tMCAO were treated with AR-R17779 (12 mg/kg) or saline ip 1 h post-surgery and thereafter once daily for the following four days. Mice were killed 7 days after surgery. (**A** and** B**) Representative micrographs of MAP2 stained coronal sections in mice treated with (A) saline and (B) α7nAChR agonist AR-R17779; scale bar corresponds to 2 mm. (**C** and** D**) Mean percentage ipsilateral injury of MAP2-stained sections in whole hemisphere (C) and in cortex (D) (MCAO *n*=13–15/group; Sham: *n*=3/group). (**E–J**) Iba1-stainings were used to assess microglial activation in (E) striatum, (F) upper cortex, (G) lower cortex, (H) cerebral peduncle, (I) globus pallidus and (J) substantia nigra (MCAO *n*=13–15/group, Sham: *n*=3/group), *c*: contralateral, *i*: ipsilateral. Data were analyzed with two-way ANOVA with operators as co-variant. Data are expressed as mean ± SEM.

To investigate the effect of the selective α7nAChR agonist AR-R17779 on stroke-induced brain injury, infarct size and microglial activation were analyzed using immunohistochemistry 7 days following tMCAO. Infarct size was calculated in paraffin brain sections stained with the neuron marker MAP2. There was no detectable difference in infarct size in mice receiving the AR-R17779 compared with saline controls, neither in whole hemisphere nor in the cortex region specifically (Figure 2A–D). Infarct size was found to not be dependent on the operator. We further characterized microglial activation in the brain by phenotype scoring in sections stained with the microglia marker Iba-1. tMCAO surgery *per se* caused microglial activation compared with mice undergoing sham surgeries (Figure 2E–J). However, we did not detect any differences in microglial activation between AR-R17779-treated mice and saline-treated mice in any of the regions (i.e. striatum, upper and lower cortex, substantia nigra, globus pallidus and cerebral peduncle) analyzed (Figure 2E–J).

### α7nAChR agonist AR-R17779 decreases white blood cell count in sham mice

**Figure 3:**
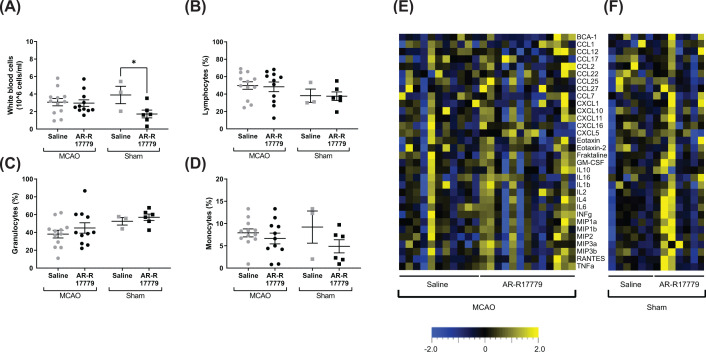
α7nAChR agonist AR-R17779 decreases white blood cell count in sham mice but does not affect hematology parameters or serum cytokines in tMCAO mice (**A–D**) Hematology analysis of blood collected at sacrifice, 7 days following tMCAO. (A) Concentration of white blood cells and percentage of (B) lymphocytes, (C) granulocytes and (D) monocytes in blood (MCAO *n*=11–12/group; Sham: *n*=3–6/group). Data were analyzed with unpaired *t*-test within each surgery group. Data are expressed as mean ± SEM, *P<0.05. (**E-F**) Heat maps showing cytokines and chemokines in serum blood at the time of euthanasia, 7 days following surgery, in mice treated with α7nAChR agonist AR-R17779 (12 mg/kg) or saline in (E) MCAO mice and (F) sham animals (MCAO *n*=11–13/group; Sham: *n*=6–7/group). Data were analyzed with Kruskal–Wallis, followed by Bonferroni correction.

Blood hematology parameters were investigated to evaluate the peripheral effects of treatment with the selective α7nAChR agonist AR-R17779. In mice undergoing sham surgery AR-R17779 significantly decreased white blood cell count compared with saline treated mice (Unpaired *t*-test, *P*=0.0497, Figure 3A). Though, there was no difference between treatment groups in mice undergoing tMCAO. In absolute numbers, a similar finding was seen in the monocyte population, with decreased monocyte count after AR-R17779 treatment in sham mice (Unpaired *t*-test, *P*=0.0446, Table 1). However, when analyzing the percentage of lymphocytes, monocytes or granulocytes there were no differences (Figure 3B–D).

**Table 1:** Hematology parameters, body weight change, brain weight and normalized spleen weight in mice undergoing tMCAO or sham surgeries with/without treatment with α7nAChR agonist AR-R17779

	MCAO		Sham	
	Saline, *n*=12–13	AR-R17779, *n*=11–14	Saline, *n*=3–6	AR-R17779, *n*=6–7
Lymphocytes (10^6^ cells/ml)	1.39 ± 0.16	1.27 ± 0.11	1.34 ± 0.16	0.70 ± 0.19
Monocytes (10^6^ cells/ml)	0.27 ± 0.06	0.21 ± 0.06	0.42 ± 0.19	0.08 ± 0.004#
Granulocytes (10^6^ cells/ml)	1.17 ± 0.23	1.49 ± 0.37	2.13 ± 0.66	0.94 ± 0.24
BW change (%)	−3.90 ± 1.53*	−0.64 ± 1.19	2.24 ± 1.25	2.65 ± 0.93
Brain weight (g)	301 ± 4.27**	302 ± 4.81	325 ± 4.35	331 ± 3.23
Normalized spleen weight (mg/10 g BW)	34.6 ± 1.8**	36.7 ± 2.2	49.1 ± 5.6	41.1 ± 2.6

Body weight (BW). Hematology parameters were analyzed with unpaired *t*-test within each surgery group. Body weight change and tissue weights were analyzed with one-way ANOVA followed by Sidak’s multiple comparisons test. Data are expressed as mean ± SEM. #*P*<0.05, Sham AR-R17779 versus Sham saline; *P<0.05, MCAO saline versus Sham saline; **P<0.01, MCAO saline versus Sham saline.

Serum samples were investigated for cytokine levels using a multiplex approach, where no differences were seen between the groups (Figure 3E–F, Supplementary Table S1). As expected, mice that underwent MCAO surgery decreased in body weight compared with control mice that underwent sham surgery (one-way ANOVA, *P*=0.0090, Sidak’s multiple comparisons test, *P*=0.0125, Table 1). Furthermore, both brain weight (one-way ANOVA, *P*=0.0001, Sidak’s multiple comparisons test, *P*=0.0069) and normalized spleen weight (one-way ANOVA, *P*=0.0063, Sidak’s multiple comparisons test, *P*=0.0024) were significantly decreased in MCAO mice compared with sham controls. Nevertheless, no difference was seen between the treatment groups (Table 1).

## Discussion

Inflammation plays a central role in stroke-induced brain injury, and the α7nAChR has been shown to modulate inflammation both in the central nervous system and in the periphery. In the present study, we investigated the expression patterns of the α7nAChR encoding gene *Chrna7* in the naïve mouse brain and evaluated the effect of the selective α7nAChR agonist AR-R17779 on stroke-induced brain injury. Although *Chrna7* was detected in all analyzed brain regions, the highest expression levels were found in cortex and hippocampus. However, treatment with AR-R17779 for five days after ischemia–reperfusion injury did not affect brain infarct size or microglial activation. Furthermore, treatment had no effect on blood hematology parameters or serum cytokine levels in tMCAO mice.

The α7nAChR is expressed by different cell types in the brain, such as neurons and microglia cells [7,14]. According to genome-wide analysis of human brain tissue in Human Protein Atlas (http://www.proteinatlas.org) [37], the highest *CHRNA7* RNA expression levels are found in cortex, hypothalamus, pons and medulla, with similar patterns in mouse brain based on RNA-seq data (https://www.proteinatlas.org/ENSG00000175344-CHRNA7/brain). In the present study, we investigated the absolute expression of *Chrna7* in brain regions known to be affected by ischemia–reperfusion injury induced by the tMCAO model, i.e. cortex, striatum, thalamus and hippocampus. Interestingly, we could confirm the expression patterns previously seen in human and mouse brain, with relatively high expression in cortex compared with striatum and thalamus. Furthermore, hippocampus displayed high expression levels compared to thalamus, which is in line with a previous study observing high *Chrna7* expression in mouse telencephalon during development [38]. Although, during normal aging only minor expressional changes of *Chrna7* have been observed in the rodent brain [39–41]. Nevertheless, gene expression does not necessarily directly translates to protein expression. In fact, some reports show post-transcriptional modifications of the *Chrna7* transcript [42,43]. Therefore, investigation of α7nAChR protein levels is needed but is currently limited by the lack of specific, commercially available antibodies [44,45]. Thus, results from RNA studies should be interpreted with caution unless careful validation can be performed on the protein level.

We and others have demonstrated an immune modulating effect of the α7nAChR in the periphery, using both genetic models [17,46–48] and pharmacological tools that stimulate the α7nAChR such as AZ6983, AR-R17779 or PNU-282987 [16,29,49,50]. This effect is predominantly mediated via α7nAChR expressed in monocytes/macrophages. Accordingly, microglia, the resident macrophage of the brain, expresses α7nAChR and can modulate an inflammatory response *in vitro* [14,51]. Given that we have previously shown an immune modulating effect of the α7nAChR agonist AR-R17779 in primary microglia cultures [14] and that AR-R17779 has not been tested in *in vivo* models of brain injury, we set out to investigate the potential *in vivo* effect of AR-R17779 to decrease neuroinflammation and counteract stroke-induced brain damage using the tMCAO model. Surprisingly, AR-R17779 treatment had no effect on brain injury or inflammatory parameters. An important distinction is that most studies investigating the effect of α7nAChR agonists and/or antagonists on brain injury have used other models, such as hemorrhagic stroke [21,52], photothrombotic stroke [20] or permanent MCAO (pMCAO) [53–55]. Nevertheless, other studies using the tMCAO model also describe an alleviation of brain injury using different α7nAChR agonists, PHA568487 [56] and PHA543613 [57] in rats and PNU282987 [58] in mice. Interestingly, in addition to data showing an immunomodulatory effect as the mechanism of action [53,56,57], studies also suggest that neurogenesis [58] and neuroprotection [52] can contribute to the beneficial effect of α7nAChR stimulation on brain injury.

The rationale for choosing the AR-R17779 in the present study was that, to the best of our knowledge, it has not previously been tested for ischemic brain injury. Moreover, this compound penetrates the blood–brain barrier [23] and, in contrast with most other α7nAChR agonists, is a full agonist and highly specific to the α7nAChR [59]. In fact, AR-R17779 shows low cross-reactivity with 5-HT3 receptors, a common feature of other α7nAChR agonists, which might explain the absence of an anti-stroke effect for AR-R17779. On the other hand, the α7nAChR antagonist MLA has been shown to increase infarct volume in both pMCAO [53,54] and tMCAO [58], further supporting a protective role for the α7nAChR. Another possible reason for the lack of effect might be that the dose of AR-R17779 was too low. However, previous studies have used AR-R17779 in concentrations in the range of 1–20 mg/kg in different rodent models, where effects on cognitive functions [60] as well as peripheral inflammation [30] have been observed. Hence, we chose a dose of 12 mg/kg based on these findings. However, a higher dose or more frequent administration after this type of severe brain damage might be needed.

In previous studies investigating α7nAChR agonists in different models of brain injury, the beneficial effect of α7nAChR stimulation were suggested to be mediated in part via decreased inflammation. Indeed, in line with previous findings where AR-R17779 decrease inflammation in other experimental models [29,30] AR-R17779 treatment decreased white blood cells and monocytes in sham mice; however, no effects were seen in tMCAO mice. Possibly, the dose and treatment regime used in the present study may be enough to modulate inflammation in mice undergoing sham surgery but not after a severe insult such as tMCAO.

## Conclusion

By using absolute quantification, we show differential expression patterns for *Chrna7* in different regions of the mouse brain, with higher expression in cortex and hippocampus. However, despite a decrease in white blood cells in sham mice treated with α7nAChR agonist AR-R17779, brain injury and microglial activation after tMCAO was not altered by AR-R17779 treatment. Possible explanations for these discrepancies between previous studies and ours can be the different properties of the agonists used and dosing regimen. Our results, together with the positive effects obtained with other α7nAChR agonists, may suggest that these agonists are better suited than AR-R17779 for the treatment of stroke-induced brain injury. However, further studies are needed to confirm these findings.

## Supplementary Material

Supplementary Figure S1 and Table S1

## Data Availability

All data are included within the main article and the supplementary files.

## Abbreviations

α7nAChR: alpha 7 nicotinic acetylcholine receptor
CCA: common carotid artery
CNS: central nervous system
DAMP: danger-associated molecular pattern
ddPCR: droplet digital PCR
ECA: external carotid artery
Iba-1: ionized calcium-binding adapter molecule 1
ICA: internal carotid artery
MAP2: marker microtubule-associated protein 2
MCA: middle cerebral artery
MCAO: middle cerebral artery occlusion
pMCAO: permanent MCAO
tMCAO: transient MCAO
tPA: tissue plasminogen activators
