# Supplementary material for: The selective alpha7 nicotinic acetylcholine receptor agonist AR-R17779 does not affect ischemia–reperfusion brain injury in mice
Source: Biosci Rep. 2021 Jun 11;41(6):BSR20210736. doi: 10.1042/BSR20210736 (PMC8200656; doi:10.1042/BSR20210736)
Supplement: Supplementary Figure S1 and Table S1 [file BSR-2021-0736_supp.pdf]

a

Score	Iba-1 immunoreactivity
0	No microglial activation
1	Focal activation
2	Mild diffuse activation, occasional amoeboid microglia
3	Widespread activation, predominant amoeboid microglia
4	Tissue loss

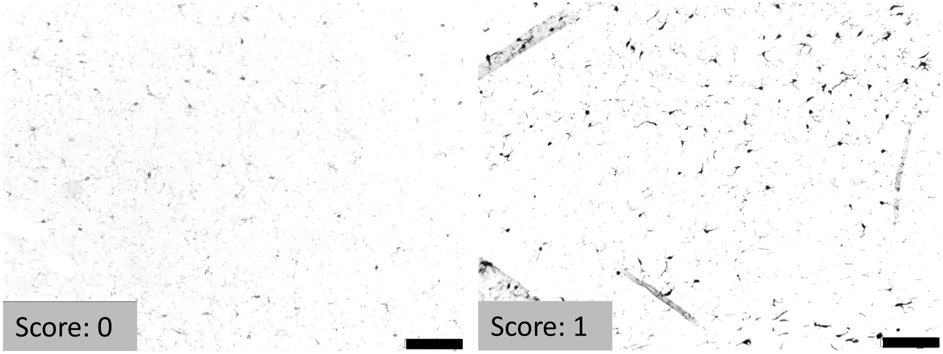

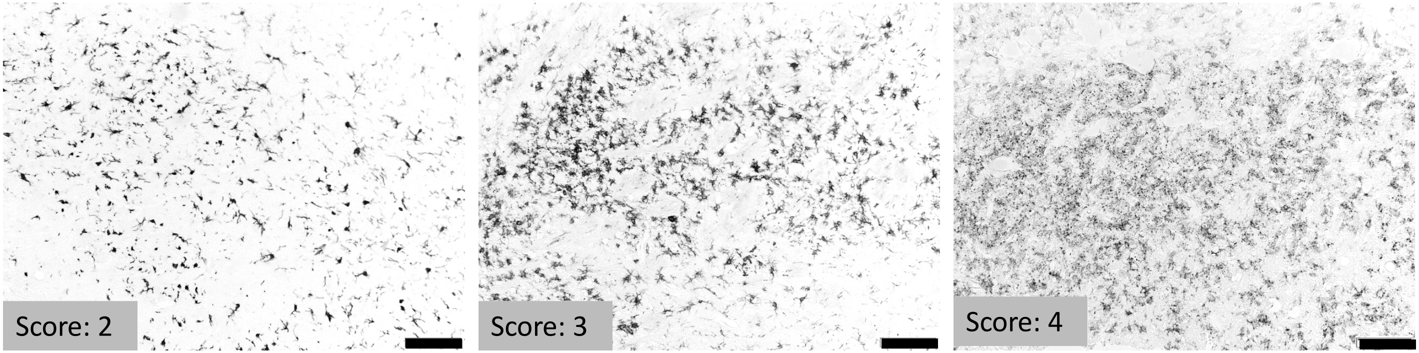

b

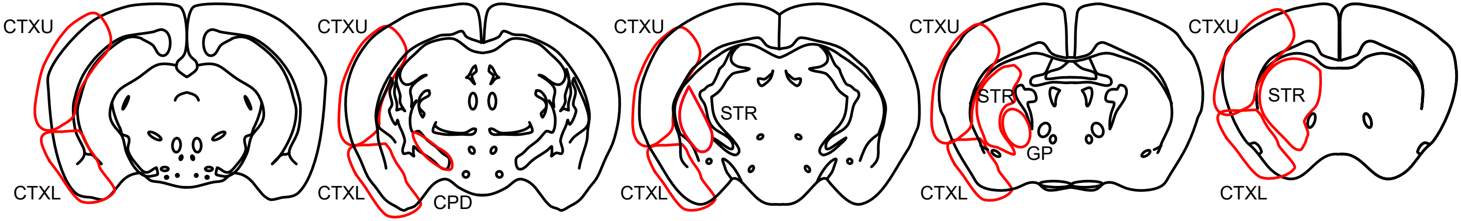

Supplementary Figure 1. (**a**) Definition of microglial scoring and representative micrographs of Iba-1 stained striatum at 20x magnification, showing different scores of microglial activation. Scale bar corresponds to 100 µm. (**b**) Pictures of coronal brain sections illustrating scored regions. CTXU: Cortex (upper), CTXL: Cortex (lower), CPD: Cerebral Peduncle, STR: Striatum, GP: Globus Pallidus

Supplementary Table 1 Serum concentration of proteins seven days after tMCAO.

Protein	MCAO Saline	MCAO AR-R17779	Sham Saline	Sham AR-R17779
**BCA-1**	9,062 (724)	10,295 (1,363)	10,713 (1,414)	11,915 (1,568)
**CCL27**	1,598 (114)	1,730 (112)	1,525 (168)	1,631 (141)
**CXCL5**	6,117 (492)	5,749 (675)	4,435 (662)	4,455 (752)
**Eotaxin**	1,456 (145)	1,648 (132)#	992 (125)	1,071 (210)
**Eotaxin2**	10,537 (977)	11,121 (540)	10,582 (1,253)	10,376 (1,134)
**Fraktalkine**	269 (10)	269 (10)	230 (10)	255 (16)
**GM-CSF**	74 (1.9)	73 (2.7)	67 (3.4)	77 (5.6)
**CCL1**	43 (2.1)	44 (1.5)	41 (6.6)	47 (4.4)
**INFg**	67 (2.3)	68 (2.4)	59 (3.7)	68 (5.5)
**IL1b**	326 (9.1)	331 (7.5)	299 (15)	309 (19)
**IL2**	36 (1.1)	38 (1.5)	33 (1.3)	37 (1.7)
**IL4**	9 (0.3)	10 (0.4)	8 (0.4)	9 (0.8)
**IL6**	75 (2.1)	74 (2.7)	69 (2.9)	78 (5.1)
**IL10**	728 (22)	718 (33)	679 (20)	679 (36)
**IL16**	540 (35)	588 (24)	532 (36)	565 (41)
**CXCL10**	1,237 (42)	1,246 (36)	1,134 (61)	1,187 (56)
**CXCL11**	656 (20)	653 (31)	561 (28)	622 (40)
**CXCL1**	130 (3.3)*	129 (3.6)†	111 (3.1)	121 (5,5)
**CCL2**	530 (8.5)	532 (18)	541 (51)	512 (32)
**CCL7**	120 (8.3)	115 (9.3)	96 (5.9)	113 (19)
**CCL12**	65 (1.8)	64 (3.4)	59 (2.1)	59 (4.4)
**CCL22**	148 (11)	149 (11)	141 (27)	133 (15)
**MIP1a**	47 (0.9)	47 (1.5)	42 (1.3)	45 (2.1)
**MIP1b**	248 (4.4)	244 (6.0)	228 (8.1)	244 (10)
**MIP2**	166 (6.6)	172 (6.8)	140 (7.4)	155 (9.1)
**MIP3a**	63 (2.0)	65 (2.2)	62 (4.8)	77 (10)
**MIP3b**	243 (6.9)	246 (7.4)	233 (11)	244 (16)
**RANTES**	90 (2.1)	97 (3.4)	83 (3.8)	94 (10)
**CXCL16**	259 (21)	269 (14)	261 (20)	272 (15)
**CXCL12**	12,362 (3,557)	19,929 (5,257)	8,700 (2,424)	34,246 (23,965)
**CCL17**	190 (13)	194 (12)	155 (24)	159 (16)
**CCL25**	3,966 (539)	4368 (605)	4754 (1142)	3453 (772)
**TNFa**	135 (5.6)	138 (8.0)	124 (13)	161 (25)

Data are compared with Kruskal-Wallis, and significance adjusted with Bonferroni correction for multiple analysis. Concentrations are presented in pg/ml as mean (SEM). *#p=0.026* MCAO AR-R17779 vs Sham Saline, **p=0.026* MCAO Saline vs Sham Saline, *†p=0.046* MCAO AR-R17779 vs Sham Saline
